# Phosphor-Doped Thermal Barrier Coatings Deposited by Air Plasma Spray for In-Depth Temperature Sensing

**DOI:** 10.3390/s16101490

**Published:** 2016-09-28

**Authors:** Di Peng, Lixia Yang, Tao Cai, Yingzheng Liu, Xiaofeng Zhao, Zhiqi Yao

**Affiliations:** 1 Key Lab of Education Ministry for Power Machinery and Engineering, School of Mechanical Engineering, Shanghai Jiao Tong University, 800 Dongchuan Road, Shanghai 200240, China; idgnep8651@sjtu.edu.cn (D.P.); caitaohust@163.com (T.C.); 2 School of Material Science and Engineering, Shanghai Jiao Tong University, 800 Dongchuan Road, Shanghai 200240, China; yanglixia20081116@126.com (L.Y.); xiaofengzhao@sjtu.edu.cn (X.Z.); 3 Gas Turbine Research Institute, Shanghai Jiao Tong University, 800 Dongchuan Road, Shanghai 200240, China; 4 Corporate Technology, Siemens Ltd. China, 7 Wangjing Zhonghuan Nanlu, Beijing 100102, China; zhiqi.yao@siemens.com

**Keywords:** phosphor, thermal barrier coating, temperature sensing, air plasma spray

## Abstract

Yttria-stabilized zirconia (YSZ)-based thermal barrier coating (TBC) has been integrated with thermographic phosphors through air plasma spray (APS) for in-depth; non-contact temperature sensing. This coating consisted of a thin layer of Dy-doped YSZ (about 40 µm) on the bottom and a regular YSZ layer with a thickness up to 300 µm on top. A measurement system has been established; which included a portable; low-cost diode laser (405 nm); a photo-multiplier tube (PMT) and the related optics. Coating samples with different topcoat thickness were calibrated in a high-temperature furnace from room temperature to around 900 °C. The results convincingly showed that the current sensor and the measurement system was capable of in-depth temperature sensing over 800 °C with a YSZ top layer up to 300 µm. The topcoat thickness was found to have a strong effect on the luminescent signal level. Therefore; the measurement accuracy at high temperatures was reduced for samples with thick topcoats due to strong light attenuation. However; it seemed that the light transmissivity of YSZ topcoat increased with temperature; which would improve the sensor’s performance at high temperatures. The current sensor and the measurement technology have shown great potential in on-line monitoring of TBC interface temperature.

## 1. Introduction

Increasing the turbine inlet temperature has been a straightforward and effective approach to improve the efficiency of power machines. For modern aero engines and gas turbines, the gas temperature at the turbine inlet is well beyond the melting temperature of the nickel-based superalloys commonly used for turbine blades and other engine components [1]. Therefore, thermal protection and cooling are usually required for safe and stable engine operation. Thermal barrier coatings (TBCs) with excellent insulation capability have been commonly used for protecting the turbine blades from excessive heat [2]. At high temperatures, a 10–15 °C increase would usually result in 50% reduction of the alloy’s lifetime [1]. Accordingly, knowing the temperature beneath the coating during engine operation is highly desirable for the evaluation of the TBC’s performance. There is no doubt that determining the temperature at the interface between the TBC and the alloys would be critically important to identify overheating areas and prevent possible blade damage. Unfortunately, it is usually not possible to use common temperature sensors in such a harsh environment (high temperature and high rotation rate). However, the non-contact measurement technique based on thermographic phosphors, which has been proved to be a viable approach for detecting surface temperature [3,4,5,6,7], could serve as a promising method in temperature sensing underneath the TBC. The principle of this technique is based on the thermal quenching of phosphorescence from inorganic materials (phosphors). Typically, a phosphor-doped coating is prepared and applied on the surface of a test object. The phosphor can be excited by a light source with short wavelength (laser, UV-LED, etc.), and its long-wavelength emission characteristics (intensity, lifetime, etc.) can be directly related to the surface temperature. The phosphor’s intensity level and lifetime usually decrease as temperature increases. More recently, phosphor thermometry based on photon up-conversion (with near IR excitation and short-wavelength emission) has also been developed [8,9,10].

The application of phosphor thermometry to TBC temperature measurement faces two major challenges: (1) the sensor material needs to be bonded seamlessly with TBCs that typically consist of yttria-stablized zirconia (YSZ), and it should have minimal effects on the mechanical properties of the TBC; (2) the measurement system needs to be designed properly, so that it can resolve the weak luminescent signals at high temperatures with the presence of strong thermal radiation, even if the sensor materials are buried beneath the TBC layer. Previous research showed that YSZ coatings doped with rare earth materials including europium (Eu), dysprosium (Dy), samarium (Sm) and erbium (Er) were suitable for TBC temperature sensing [11,12]. Those phosphors have shown strong luminescent signals and excellent temperature sensitivity over a wide range of temperatures up to 1100 °C [5]. The use of other phosphors, such as Y_2_O_3_:Eu [13] and YAG:Dy [14], has also been attempted for the same purpose. The TBC coatings are commonly prepared by air plasma spray (APS, generally employed for heavy duty gas turbines) or by electron beam physical vapor deposition (EBPVD, generally employed for aero-engines).

TBC temperature sensing attempts using phosphors were first made for EBPVD-TBCs, since the coatings typically have columnar microstructure with relatively high light transparency to allow in-depth temperature measurements [15]. Feist et al. demonstrated temperature sensing capability up to about 700 °C using Dy-doped YSZ prepared by EBPVD [14]. Gentleman et al. conducted temperature measurements up to 1100 °C at a TBC/bondcoat interface using Eu-doped YSZ with topcoats of 127 and 187 µm [16]. Steenbakker et al. developed a multi-layer EBPVD-TBC, which contained a top YAG:Tm coating, a 100 µm undoped YSZ layer, and a Dy-doped YSZ layer at bottom [17]. The temperature sensing ranges for the top and bottom layers were 1000–1300 °C and 400–950 °C, respectively. Compared to EBPVD-TBCs, coatings deposited by APS typically have parallel structure formed by the “splat”, which increases light scattering and reduces light transmission [15]. Therefore, it is extremely challenging to collect sufficient luminescent signal for in-depth measurements of APS-TBC at high temperatures. The sensing range is expected to be limited for a thick topcoat due to the signal attenuation by light scattering and absorption. Chen et al. first attempted temperature sensing of APS-TBC using Dy-doped YSZ without any topcoats, and achieved an upper sensing limit over 900 °C [18]. A subsequent work by Feist and Heyes demonstrated in-depth temperature sensing up to 800 °C with a topcoat as thick as 500 µm [19]. They used a pulsed Nd:YAG laser with a third harmonic generator to produce 355 nm light (at 16 Hz) as the excitation source. Interestingly, according to their results the topcoat thickness had no obvious effects on the temperature sensing range. More recently, a coating system for TBC temperature sensing were applied on a Rolls-Royce jet engine by Feist et al., but no in-depth measurement was reported [20].

In the current study, Dy-doped TBCs were prepared by APS for in-depth temperature sensing using phosphor thermometry. The pulsed excitation was provided by a 405 nm, 5 W diode laser instead of a 355/532 nm Nd:YAG laser commonly used in previous studies [16,17,18,19]. This portable, low-cost laser could be modulated up to 30 kHz in comparison to 10–20 Hz for a Nd:YAG laser. This greatly improved the overall sampling rate, especially at high temperatures where the average of multiple measurements is usually required for achieving sufficient SNR. Temperature sensing properties were examined for the TBC samples with different thickness values of the YSZ topcoat (from 0 to 300 µm). The effects of topcoat thickness on the luminescent signal attenuation have also been investigated in detail. This in-depth sensing technology provided basis for the development of a multi-layer heat flux sensor. It is possible to dope the top and bottom of TBC layer with different phosphors and directly obtain the temperature difference and the heat flux across the TBC.

## 2. Fabrication Method and Properties of Phosphor-Doped TBC 

### 2.1. Materials and Fabrication Method

The phosphor powders were prepared by the reverse co-precipitation method, which was selected to avoid the segregation of the cations and ensure complete molecular mixing between the dopant and the host material. The typical procedures for sample synthesis are described as follows: (1) Dy_2_O_3_ and Y_2_O_3_ were weighed according to the target composition, and were dissolved in nitric acid under heating; (2) after the Dy_2_O_3_ and Y_2_O_3_ were completely dissolved, the excess nitrite acid was removed at an elevated temperature; (3) ZrOCl_2_·8H_2_O and deionized water were added to obtain a mixture solution which was then magnetically stirred for 24 h; (4) the mixed solution was slowly dropped into aqueous ammonia solution, and the precipitate was filtered, washed and dried in air for 24 h; (5) the obtained powder was sifted and calcined at 950 °C for 12 h; (6) then the powder was pressed into tablets by a uniaxial cold pressing under 100 MPa and then sintered at 1500 °C for 4 h in air; (7) the final powder product was granulated to improve its mobility for plasma spray.

The Dy-doped YSZ and the regular YSZ coatings were deposited using a plasma spraying system. This 80 kW APS system used argon as the primary gas, and hydrogen as the secondary gas. The carrier gas was argon with a particle velocity over 300 m/s during operation. Initially, a Hastalloy substrate was blasted using alumina particles to obtain an initial roughness of 6–10 μm. Then the 150 μm thick NiCoCrAlY bond coat was deposited onto the substrate. The Dy-doped YSZ was sprayed on top of the substrate, and then the regular YSZ coating was sprayed on top of the phosphor-doped layer. The thickness of the YSZ topcoat was varied by applying different number of sample sweeps during APS. Please note the Dy-YSZ layers in different samples were deposited from one spray to ensure relatively constant coating thickness and phosphor concentration. This allowed direct comparison between different samples to investigate the effects of the topcoat thickness.

### 2.2. Coating Properties

**Figure 1:**
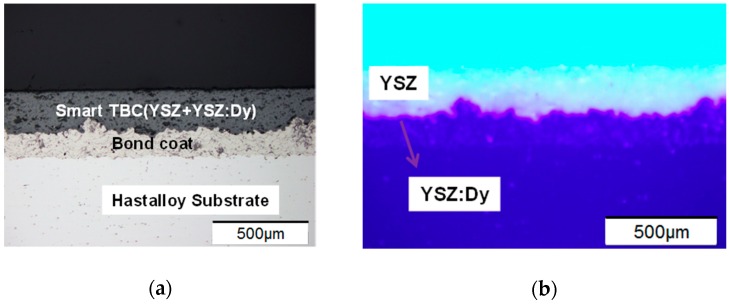
Microstructure of phosphor-doped TBC: (**a**) no excitation and (**b**) excited by UV-LED.

The microstructure of the phosphor-doped TBC sample was examined using micro metallographic microscope (SZ61, Olympus, Tokyo, Japan), as shown in Figure 1a. The interfaces between the Hastalloy substrate and the bond coat, and between the bond coat and the TBC can be clearly seen. Here, the YSZ serves as the thermal barrier layer and YSZ:Dy serves as the temperature sensing layer.

**Figure 2:**
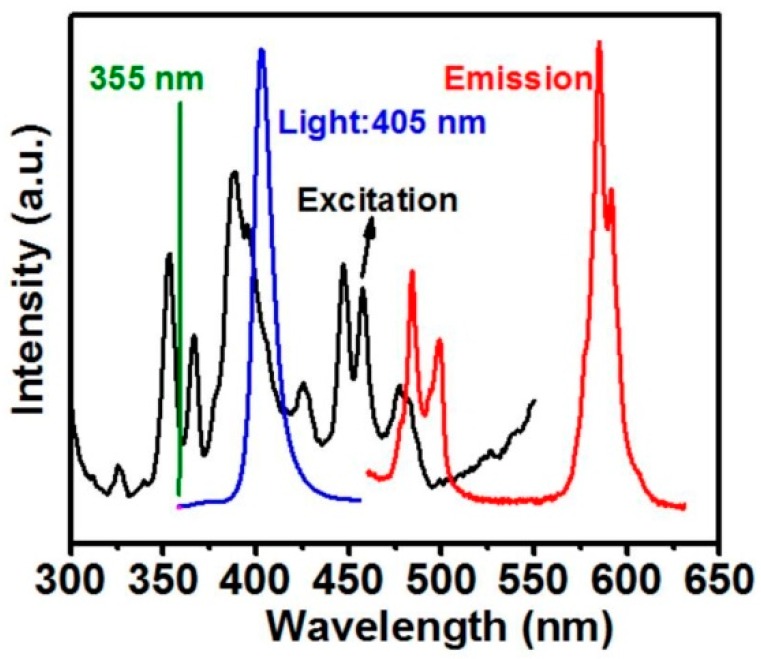
Absorption and emission spectrum of YSZ:Dy.

The microstructure of the sample was also examined under the excitation of a 405 nm diode laser (OEM-HD-405, CNI Laser, Changchun, China) to clearly show the sensing layer, which is indicated by the purple ribbon under the white YSZ in Figure 1b. In the current study, the thickness of the YSZ:Dy layer was about 40 μm for all samples. A total of four samples were made with topcoat thicknesses of 0, 80, 180 and 300 μm. The Raman spectrum of Dy-doped YSZ was examined using a Raman spectrometer (LabRAM HR Evolution, Horiba, Kyoto, Japan). Figure 2 shows the absorption spectrum (in black) and the emission spectrum (in pink) of YSZ:Dy. The strongest absorption peak appears at 390 nm, and the strongest emission peak occurs at 584 nm. Previous studies have commonly used the 355 nm Nd:YAG laser as exciting source for YSZ:Dy, which is actually less efficient then the 405 nm light source being used in the current study. Based on the emission spectrum, a band-pass filter of 550–625 nm was selected for the collection of the luminescent signal.

## 3. Temperature Sensing Method

### 3.1. Fundamentals

**Figure 3:**
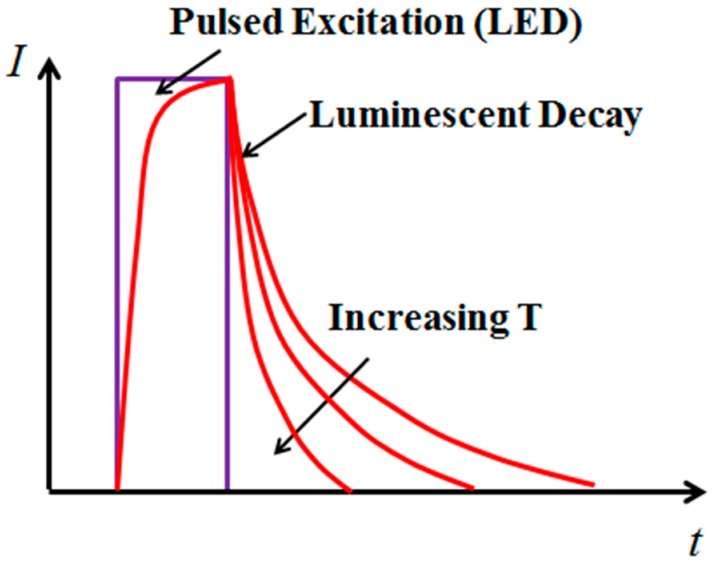
Principle of lifetime-based temperature sensing using phosphors.

The response of a phosphor to a pulsed excitation is typically a single-exponential decay, which can be expressed as:

(1) $$I=I_{0}\cdot e^{-t/\tau }$$

where *I_0_* is the intensity at the start of the decay, and *τ* is the lifetime of the phosphor. The lifetime τ was found to be very sensitive to temperature within a certain range for each phosphor, and was therefore suitable for temperature sensing [3,4]. In the current study, the excitation pulse was provided by a 405 nm diode laser, and the luminescent decay lifetime reduced as the temperature increased, as shown in Figure 3. The luminescent decay signal *I*(*t*) was recorded using a PMT to obtain *τ*, which was directly related to the temperature through calibration. A standard approach for calculating *τ* is to apply a linear least-square fit on the logarithm of the intensity (log(*I*(*t*))). It should be noted that the phosphor’s response can take the form of a multi-exponential decay due to the energy transfer process. For example, Eu-doped YSZ coating was found to have a bi-exponential decay with a fast decay constant *τ_1_* and slower decay constant *τ_2_* at high temperatures [16,21]. However, YSZ:Dy has shown clear features of a single-exponential decay within the temperature range in the current study. Therefore, Equation (1) has been used for the lifetime determination.

### 3.2. Measurement System

**Figure 4:**
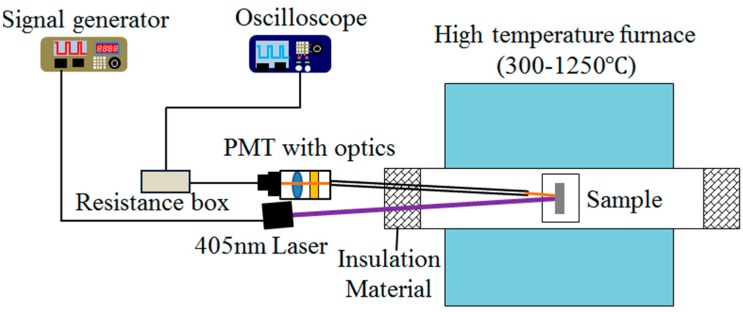
Schematic of TBC temperature sensing system.

The schematic diagram of the temperature sensing system using phosphor-doped TBC is shown in Figure 4. The TBC sample was placed in a high temperature tube furnace (SGL-1400, SIOMM, Shanghai, China) with a working temperature range from 300 to 1250 °C and ±2 °C accuracy. The sample was excited by the 405 nm diode laser being modulated by a function generator (AFG1022, Tektronix, Beaverton, OR, USA). The luminescent signal first went through a sight tube and was then collected by a PMT module (h9305-03, Hamamatsu, Shizuoka, Japan) through a camera lens (60 mm, f/2.8, Nikon, Tokyo, Japan). A band-pass filter (550–625 nm) was installed before the camera lens to exclude both the excitation light and the thermal radiation at high temperatures. The current signal from the PMT was converted to voltage through a resistance box (R = 1000 Ω) and then recorded by an oscilloscope (Tektronix DPO2002B) with a sampling rate of 3.125 MHz. For each measurement, the PMT signal was averaged over 512 samples. A K-type thermocouple (XC-K-14-25, 0–1090 °C, ±1%, Omega, Stamford, CT, USA) was placed close to the TBC sample to monitor the temperature during the calibration. Insulation material (asbestos) was placed on both ends of the tube to improve the temperature uniformity within the furnace.

### 3.3. Lifetime Determination

Generally, the determination of the luminescent lifetime includes three steps: the background subtraction, the intensity normalization and the linear curve-fitting. The background signal level was evaluated using the average of 10,000 data points immediately before the start of the excitation pulse. This background value (*I_BG_*) was subtracted from the intensity values during the luminescent decay. Then all decay data were normalized by the intensity value (*I_0_*) at the end of the excitation pulse (the average of 100 data points immediately before the end of excitation). Finally, a linear curve-fit was applied to the logarithm of the normalized decay data. The uncertainty in the lifetime measurement was evaluated based on the 95% confidence range of the curve-fit.

## 4. Results and Discussion

### 4.1. Calibration Results

**Figure 5:**
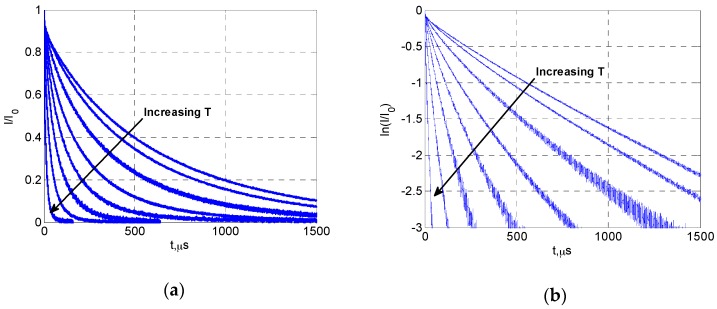
Decay curves of YSZ:Dy at different temperatures: (**a**) normalized intensity; (**b**) logarithm of normalized intensity.

**Figure 6:**
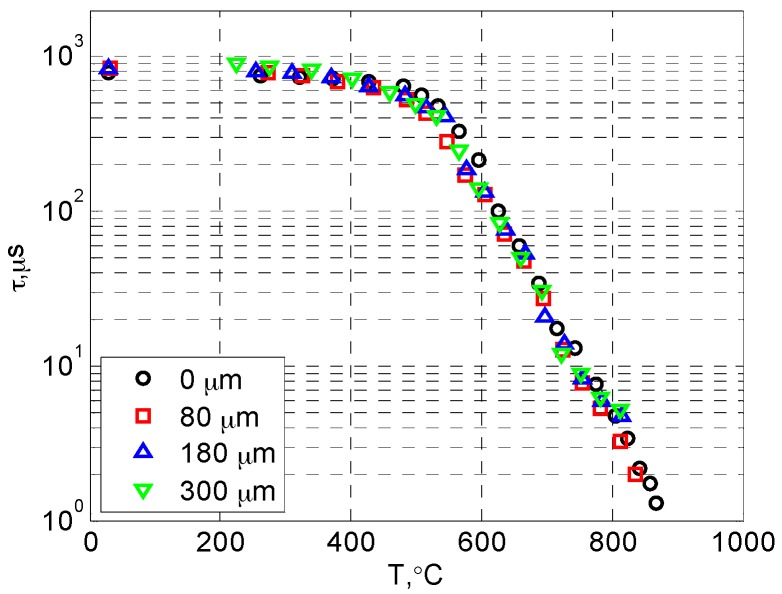
Calibration results of Dy-doped TBC with different topcoat thickness.

Figure 5 presents the luminescent decay curves of YSZ:Dy at different temperatures (from 400 to 750 °C). The decay lifetime decreases rapidly as temperature rises, and linear relations are clearly seen between *t* and *log*(*I*/*I_0_*), showing the feature of a single-exponential decay. The lifetime values were obtained from the decay curves using a linear curve-fit as mentioned earlier. The temperature calibration results from the room temperature up to about 900 °C are presented for all samples in Figure 6. It can be seen that the luminescent lifetime did not change with the topcoat thickness, as expected. The lifetime value was around 800 µs at room temperature, and it showed weak dependency on temperature from room temperature to about 500 °C (*τ* ≈ 500 µs at 500 °C). Then the temperature sensitivity suddenly increased, and a linear relation was observed between *T* and log(*τ*). The lifetime dropped to about 5 µs at 800 °C. The current results of Dy-YSZ agree well with the data from previous studies [18,19].

**Figure 7:**
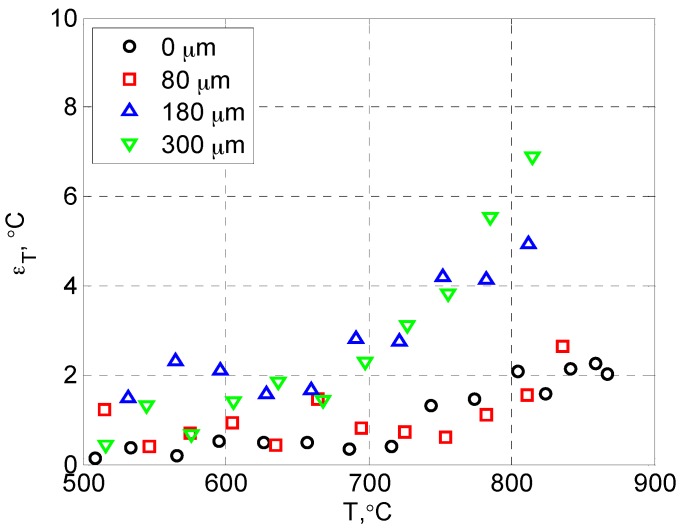
Calibration uncertainty of Dy-doped TBC with different topcoat thickness.

The measurement uncertainties within the effective sensing range (*T* > 500 °C) are presented separately in Figure 7 for clarity. The uncertainty was relatively constant for *T* < 700 °C, and then increased rapidly with temperature due to the reduced signal level (strong thermal quenching) at high temperatures. Also, the uncertainty generally increased with the topcoat thickness due to stronger signal attenuation (from light scattering and absorption) for both the excitation and the emission light as they travelled through the top YSZ layer. As a result, the upper limit of temperature sensing was reduced as the topcoat thickness increased.

### 4.2. Effects of Topcoat Thickness on the Signal Level and the Temperature Sensing Limit

The effects of topcoat thickness (APS-TBC) on temperature sensing were previously investigated by Feist and Heyes, and they found that the topcoat thickness did not affect the sensing limit. However, our results have shown a clear drop in the upper sensing limit for samples with thicker topcoats. To collaborate our finding, the effects of topcoat thickness on the luminescent signal level were also examined. Here, the signal level was evaluated using the ratio between the initial intensity at the start of decay (*I_0_*) and the background signal level (*I_BG_*). Since the luminescent signal decreases as temperature increases due to thermal quenching and the thermal radiation increases with temperature, the luminescent signal becomes small compared to the background signal (mostly thermal radiation) at high temperatures and the measurement accuracy is limited. Therefore, this signal-to-background ratio (SBR) is directly related to the upper limit of temperature sensing.

**Figure 8:**
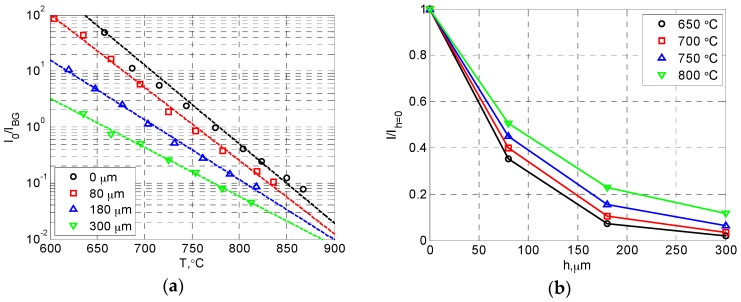
Effects of YSZ topcoat thickness on SBR: (**a**) temperature vs. SBR; (**b**) topcoat thickness (*h*) vs signal attenuation (*I*/*I_h_*_=0_).

The relations between the temperature and the SBR for all samples are presented in Figure 8a. Generally, there is a linear relation between *T* and log(*I_0_* /*I_BG_*). This is not surprising considering that the luminescent intensity decreases exponentially with temperature within this range, and the thermal radiation increases exponentially with temperature (Wien’s radiation law). At high temperatures, the low SBR values resulted in significant measurement error (±7 °C uncertainty for a SBR value of 0.05). Eventually, the SBR value would be too low to provide a reliable temperature measurement. As for the effects of topcoat thickness, it is clear that the SBR reduces as the topcoat thickness increases at a certain temperature. This indicates that samples with thicker topcoats produce less luminescent signal, since the background level should be constant at the same temperature. For example, a topcoat of 80, 180, and 300 μm caused signal attenuation of 50%, 77% and 88% at 800 °C, respectively. The results have confirmed that the topcoat thickness strongly affects the luminescent signal level, and thus the temperature sensing limit.

The linear fitted curves between *T* and log(*I_0_*/*I_BG_*) are also presented in Figure 8a. It is interesting to see that the slope of the curve decreases as the topcoat thickness increases, which indicates that there was actually less signal attenuation due to the topcoat at higher temperatures. As shown in Figure 8b, the amount of signal attenuation reduces as the temperature increases for all samples with a topcoat. A plausible explanation is that the light transmissivity of the YSZ coating increases with temperature, which actually benefits the phosphor-based TBC temperature sensing. This may also be part of the reason that Feist and Heyes did not observe a clear difference in the temperature sensing limit between samples with different topcoat thickness values.

## 5. Conclusions

A remote temperature sensing technology has been developed for APS TBC based on phosphor thermometry. The YSZ has been successfully integrated with dysprosium oxides to form an optical sensor. The Dy:YSZ sensing layer can be deposited beneath the regular YSZ layer by APS to perform in-depth temperature measurement. The sensing layer was excited by a 405 nm diode laser, and the luminescent decay following the laser pulses was collected by a PMT. The luminescent lifetime has shown excellent sensitivity with respect to the temperature for *T* > 500 °C. At higher temperatures, the measurement errors increased due to weaker luminescent signal and stronger thermal radiation. The upper sensing limit was eventually determined by the signal-to-background ratio (SBR), which was close to 900 °C in the current study. It was found that the existence of the YSZ topcoat caused significant signal attenuation due to light scattering and absorption. Therefore, the temperature sensing range was further limited for a thicker YSZ topcoat. However, it was very likely that the light transmissivity of the YSZ topcoat increased with temperature, which would provide benefits to the measurement at high temperatures.

For the next step, a multi-layer sensor will be developed including one sensing layer at the TBC interface and another sensing layer on the TBC surface to monitor both the temperatures and the heat flux. Also, due to the small size, the low cost and the high modulation frequency of a diode laser, it was quite possible to develop an integrated sensor system for the on-line monitoring of TBC temperature. This will be the focus of future work.

## Abbreviations

The following abbreviations are used in this manuscript:

APS	Air plasma spray
EBPVD	Electron beam physical vapor deposition
PMT	Photo-multiplier tube
SBR	Signal-to-background ratio
SNR	Signal-to-noise ratio
TBC	Thermal Barrier Coating
YSZ	Yttria-stabilized zirconia
